# Aquaporin-4 antibody testing: direct comparison of M1-AQP4-DNA-transfected cells with leaky scanning versus M23-AQP4-DNA-transfected cells as antigenic substrate

**DOI:** 10.1186/1742-2094-11-129

**Published:** 2014-07-29

**Authors:** Sven Jarius, Friedemann Paul, Kai Fechner, Klemens Ruprecht, Ingo Kleiter, Diego Franciotta, Marius Ringelstein, Florence Pache, Orhan Aktas, Brigitte Wildemann

**Affiliations:** 1 Molecular Neuroimmunology, Department of Neurology, University of Heidelberg, Heidelberg, Germany; 2 Department of Neurology Charité-Universitätsmedizin, NeuroCure Clinical Research Center and Clinical and Experimental Multiple Sclerosis Research Center, Berlin, Germany; 3 Institute for Experimental Immunology, affiliated to Euroimmun AG, Luebeck, Germany; 4 Department of Neurology, Charité University Medicine, Berlin, Germany; 5 Department of Neurology, University of Bochum, Bochum, Germany; 6 IRCCS, C. Mondino National Neurological Institute, Pavia, Italy; 7 Department of Neurology, Medical Faculty, Heinrich-Heine-University, Düsseldorf, Germany; 8 Molecular Neuroimmunology, Department of Neurology, Otto Meyerhof Center, Im Neuenheimer Feld 350, 69120 Heidelberg, Germany

**Keywords:** neuromyelitis optica, neuromyelitis optica spectrum disorders, Devic syndrome, Devic’s syndrome, NMO-IgG, antibodies to aquaporin-4, cell-based assay, M1 aquaporin-4, M23 aquaporin-4, antibody testing, longitudinally extensive transverse myelitis, optica neuritis

## Abstract

**Background:**

Neuromyelitis optica (NMO, Devic syndrome) is associated with antibodies to aquaporin-4 (NMO-IgG/AQP4-Ab) in the majority of cases. NMO-IgG/AQP4-Ab seropositivity in patients with NMO and its spectrum disorders has important differential diagnostic, prognostic and therapeutic implications. So-called cell-based assays (CBA) are thought to provide the best AQP4-Ab detection rates.

**Objective:**

To compare directly the AQP4-IgG detection rates of the currently most widely used commercial CBA, which employs cells transfected with a full-length (M1)-human AQP4 DNA in a fashion that allows leaky scanning (LS) and thus expression of M23-AQP4 in addition to M1-AQP, to that of a newly developed CBA from the same manufacturer employing cells transfected with human M23-AQP4-DNA.

**Methods:**

Results from 368 serum samples that had been referred for routine AQP4-IgG determination and had been tested in parallel in the two assays were compared.

**Results:**

Seventy-seven out of 368 samples (20.9%) were positive for NMO-IgG/AQP4-Ab in at least one assay. Of these, 73 (94.8%) were positive in both assays. A single sample (1.3%) was exclusively positive in the novel assay; three samples (3.9%) were unequivocally positive only in the ‘classic’ assay due to high background intensity in the novel assay. Both median fluorescence intensity and background intensity were higher in the new assay.

**Conclusions:**

This large study did not reveal significant differences in AQP4-IgG detection rates between the ‘classic’ CBA and a new M23-DNA-based CBA. Importantly, our results largely re-affirm the validity of previous studies that had used the ‘classic’ AQP4-CBA to establish NMO-IgG/AQP4-Ab seropositivity rates in NMO and in a variety of NMO spectrum disorders.

## Introduction

Neuromyelitis optica (NMO) is an often severely disabling syndrome characterized by optic neuritis (ON) and myelitis [1-4]. In 2004, Lennon and colleagues described a novel IgG serum reactivity present in around 60 to 80% of patients with NMO (termed NMO-IgG) [5,6], which was subsequently shown to target aquaporin-4 (AQP4), the most abundant water channel in the central nervous system (CNS) [7,8]. In the meantime, AQP4-IgG have been demonstrated to be directly pathogenic and of high differential diagnostic and prognostic impact [9-13], classifying seropositive NMO as part of an expanding spectrum of humorally mediated autoimmune syndromes of the CNS [14-21]. AQP4-IgG were also demonstrated to confer a high risk of conversion into NMO in patients presenting with a first attack of myelitis or ON [1,22-24]. Moreover, some studies suggested that AQP4-IgG seropositivity might be associated with a more severe disease course in patients with NMO [1,25]. Most importantly, however, the presence of AQP4-IgG permits differentiation between NMO and multiple sclerosis - two conditions that can be difficult to distinguish on clinical grounds and the optimum treatments of which differ - by means of a laboratory test [26-31].

Over the past couple of years, several immunoassays for the detection of AQP4-IgG have been developed, which vary significantly with regard to sensitivity, specificity and reproducibility (see [28] for a comprehensive overview). Currently, so-called cell-based assays (CBAs), most of which employ HEK293 cells transfected with human AQP4, are considered to provide the best compromise between assay performance and practical feasibility. CBAs have been shown to be both more sensitive and more specific than immunohistochemistry, the previous gold standard, enzyme-linked immunosorbent assays and some immunofluorescence assays [26-30,32,33].

At least two isoforms of human AQP4 exist. Recently, it has been suggested that the shorter M23 isoform might be preferential with regard to assay sensitivity. It has been argued that AQP4-IgG might partly target epitopes formed upon the formation of M23-AQP4 to so-called orthogonal arrays of particles (OAPs) [34]. In fact, a recent study has demonstrated higher AQP4-IgG binding affinity to M23-AQP4 than M1-AQP4 [35].

The currently most widely used commercial CBA employs cells transfected with so-called M1-AQP4-DNA. In four previous studies on European and North American patients and control subjects by three independent groups, this assay yielded median sensitivity of 78.6% (N = 103) and specificity of 100% (N = 322), corresponding to a positive likelihood ratio of ∞ and a negative likelihood ratio of 0.21 (CI 95% 0.14 to 0.3) [26,32,36,37]. From both a clinical and a scientific point of view, it would be important to know whether the data on AQP4-IgG frequencies obtained using that assay in the past are valid or if they represent an underestimate caused by the use of M1- instead of M23-AQP4-DNA. Of note, however, the sequence of the construct used by the manufacturer in that ‘classic’ assay contains a C at position -3, which has recently been shown by Pisani *et al*. to result in strong co-expression of M23 due to leaky scanning (LS); this might well compensate for the use of M1 DNA [38,39]. In the present study we directly compared the detection rates of the ‘classic’ M1-DNA-based CBA with LS and of an M23-DNA-based CBA newly developed by the same manufacturer. To this end, we evaluated results from 368 sera tested in parallel in the two CBAs.

## Patients and methods

**Figure 1:**
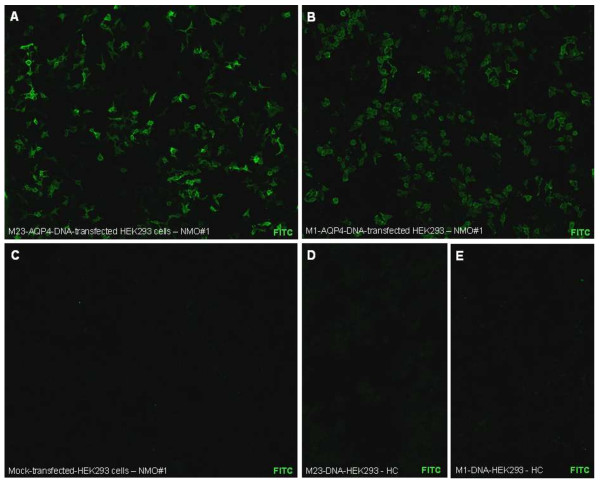
**Neuromyelitis optica (NMO)-IgG/AQP4-Ab as detected by an M23-DNA-AQP4-based, cell-based assay (CBA) and an M1-AQP4-DNA-based CBA with leaky scanning (LS).** M23-AQP4-DNA-transfected cells **(A)**, M1- AQP4-DNA-transfected cells with LS **(B)** and mock-transfected cells **(C)** were incubated in the same well on separate biochips. Note the higher signal intensity observed in the M23-AQP4-DNA-based assay in a direct comparison with the M1-AQP4-DNA-based assay observed with this particular sample (NMO#1). Panel **D** and **E** show results obtained with serum from a healthy control donor. Photographs were taken with a Nikon Ni-E upright, wide-field, research microscope using identical exposure times and camera settings. Binding of NMO-IgG/AQP4-Ab was visualized using a FITC-labeled anti-human IgG antibody. FITC = fluorescein isothiocyanate; HC = healthy control.

Slides with five wells each containing biochips coated with formalin-fixed M1-AQP4-transfected HEK293 cells (sense primer ATACGTCTCAAGCTTATGAGTGACAGACCCACAGCAAGGCGGTG and reverse primer ATACGTCTCCTCGAGTCATACTGAAGACAATACCTCTCCAGATTGGTC; [26]), M23-AQP4-transfected HEK293 cells (sense primer ATAAGGTCTCCCATGGTGGCTTTCAAAGGGGTCTGGAC and reverse primer ATACGTCTCCTCGAGTCATACTGAAGACAATACCTCTCCAGATTGGTC) and mock-transfected HEK293 cells were obtained from Euroimmun (Luebeck, Germany). Correctness of the AQP4-encoding DNA was verified by sequencing by the manufacturer. For standardization of the immunological analyses, the TITERPLANE™ technique was used according to the manufacturer’s instructions as described [26]. In short, samples (at standard 1:10 dilution in 1% BSA in PBS) or labeled antibodies were applied to the reaction fields of a reagent tray. The biochip slides were then placed into the recesses of the reagent tray, where all biochips of each slide came into contact with the fluids, and the individual reactions commenced simultaneously. After incubation for 60 min at room temperature, the slides were rinsed with a flush of PBS-Tween and incubated in PBS-Tween for at least 5 min. Bound IgG were labeled using fluorescein-conjugated goat anti-human IgG antibody for 60 min and washed as described before. All sera were analyzed by the same assessor (10 years’ experience in indirect immunofluorescence assaying and 5 years’ experience with the M1-AQP4 CBA used here), who was unaware of patients’ diagnoses. Typical findings are shown in the Figure 1. Sera were classified as positive in assay A (M1-DNA-based CBA with LS), positive in assay B (M23-DNA-based CBA), or positive in both assays as previously described [26]. In addition, all positive samples were semiquantitatively scored based on the intensity of the surface staining using a five-point scale (1: very weak staining, may require 20x magnification for affirmation; 2: weak; 3: moderate; 4: strong; and 5: extraordinarily bright staining, fluorescence may overshine parts of the cytoplasm).

AQP4-IgG test results in 368 unselected serum samples from 341 subjects (median age 44 years, range 1 to 92) that had previously been referred and tested in parallel in the two assays for clinical routine purposes were retrospectively analyzed in a strictly anonymized fashion as part of an internal quality audit, i.e. without access to patient names or other identifiers. No samples were taken for the purpose of this study. Statistical analysis was performed using Microsoft Excel and GraphPad Prism. In addition, two samples that had previously yielded a negative result in another, independent C^-3^-M1-AQP4-DNA-based assay [40] but a positive result in its M23-DNA-based counterpart were retrospectively tested in the two assays; testing was approved by the institutional review board of the University of Heidelberg and patients gave informed written consent.

## Results

Seventy-seven out of 368 samples (20.9%) were positive for AQP4-IgG in one of the two assays. Testing in assay B resulted in stronger surface immunofluorescence intensity scores than in assay A in a direct comparison of biochips within the same well in the majority of cases (see the Figure 1 for an example). Seventy-three out of 77 samples (94.8%) were positive in both assays; only one sample (1.3%) was positive exclusively in assay B (fluorescence intensity score 1; diagnosis indicated by the referring center: ‘NMO according to Wingerchuk’s 2006 criteria’); three samples (3.9%) were exclusively positive in assay A (fluorescence intensity scores: one in two cases, two in one case; 1 × ‘NMO according to Wingerchuk’s 2006 criteria’, one × ‘myelitis and ON’, but no clinical information provided by the referring center on whether Wingerchuk’s 2006 criteria were met, and one × ‘myelitis’) due to higher background fluorescence observed in the M23-DNA-based cells, which prevented unequivocal distinction between AQP4-specific and non-specific fluorescence in these particular cases; 291 samples were negative both in assay A and in assay B (Table 1). Seropositivity rates did not differ significantly between assay A (20.7%) and assay B (20.1%) (*P* = n.s., Fisher exact test). Cohen kappa, as a measure of interassay reliability, was 0.967 (CI 95% 0.934 to 0.999). Thirty-eight samples yielded higher signal intensity scores in assay B; no significant difference in signal intensity was found in 27 cases; and 12 samples yielded higher scores in assay A. Median signal intensity among AQP4-IgG-positive samples was 4 (range, 1 to 5) in assay B compared to 3.5 (range 1 to 5) in the assay A at a 1:10 dilution (*P* <0.002; Mann-Whitney U test). The median difference in signal intensity score was 1 (range 1 to 3); with a difference of 1 in 40 cases, of 2 in 9 cases, and of 3 in 1 case. Very weak or weak staining (fluorescence intensity (FI) scores 1 or 2) was observed with 14 AQP4-IgG-positive samples using assay A but with only 7 using assay B; in contrast, maximum signal intensity (FI score 5) was noted with 20 AQP4-IgG-positive samples using assay, B but only with 6 AQP4-IgG-positive samples using the assay A (*P* <0.005; Chi square test; n = 77). The two samples that had previously yielded a positive result in an independent M23-DNA-based CBA but a negative result in its C^-3^-M1-DNA-based counterpart [40] and were tested in addition yielded a positive result in both assays (sample 1: FI score 3 in assay A, FI score 4 in assay B; sample 2: FI score 4 in assay A, FI score 5 in assay B).

**Table 1:** Neuromyelitis optica **(NMO)-IgG/AQP4-Ab seropositivity rates as found in an M1-AQP4-DNA-based cell-based assay with leaky scanning (LS, ‘assay A’) and in an M23-AQP4-DNA-based cell-based assay (’assay B’)**

**NMO-IgG/AQP4-Ab**^**a**^	
Positive	
Either assay A or B	77/368 (20.9%)
Assay A	76/368 (20.7%)
Assay B	74/368 (20.1%)
Both assay A and B	73/77 (94.8%)
Assay A only	3/77 (3.9%)
Assay B only	1/77 (1.3%)
Negative	
Neither assay A nor B	291/368 (79.1%)

^a^M1-DNA- and M23-DNA-transfected cells were tested simultaneously on separate biochips within the same well to ensure identical incubation conditions.

## Discussion

In the present study, one of the largest on NMO-IgG/AQP4-IgG testing so far (n = 368), we found no significant difference in positivity rates between the currently most widely used commercial CBA, which employs HEK293 cells transfected with a construct based on C^-3^-M1-AQP4-DNA allowing for LS, and a newly developed M23-DNA-based CBA from the same manufacturer, despite higher median signal intensity in the new assay. Importantly, M1- and M23-AQP4-transfected cells were incubated in the same well and thus analyzed simultaneously under identical conditions. Most notably, only a single sample was positive exclusively in the novel M23-AQP4-DNA-based assay.

This is clinically important given that (a) the M1-DNA-based (‘classic’) assay evaluated here has been used by many laboratories over the past couple of years and employed in many scientific studies on NMO and its spectrum disorders, and (b) some recent studies have suggested that transfection with the shorter, so-called M23 isoform of AQP4 might improve assay sensitivity. That latter assumption is corroborated by preliminary evidence suggesting that AQP4-Ab may partly bind to conformational epitopes linked to OAP formation or that larger OAPs could enhance NMO-IgG/AQP4-Ab binding [34,35,40,41].

There are at least two possible explanations between the hypothesis that transfection with M23-AQP4 is preferential in terms of sensitivity [34] and the finding of almost equal sensitivity in practice as observed in the present and in previous studies. First, NMO patients may simply harbor not only M23-specific AQP4-IgG in their serum but generally also an amount of AQP4-IgG binding to M1-AQP4, or both M1-AQP4 and M23-AQP4, sufficient to yield positive test results also in M1-based assays. In fact, recent affinity studies using AQP4-transfected human astrocyte-derived U87MG cells found binding to both isoforms, though consistently stronger binding to M23 with wide variations in NMO-IgG/AQP4-Ab binding intensity to M1- versus M23-AQP4 among patients and even among recombinant monoclonal AQP4-Abs generated from different plasma cell clones of a single patient [35]. Second, and importantly, Pisani *et al*. recently demonstrated that LS-induced by a C or T in position N^-3^ may result in substantial expression of M23-AQP4 in HEK293 cells even if M1-AQP4-DNA is employed [38,39]. Given that the sequence employed in the M1-AQP4-DNA-based CBA used in our study in fact contains a C at position N^-3^, expression of M23 due to LS is indeed a likely explanation for our finding of equal AQP4-IgG detection rates of the two assays. Future studies employing full-length AQP4-DNA should pay special attention to vector preparation and possible LS.

However, other factors may also play a role, as indicated by the fact that samples from our laboratory that had been found negative in another C^-3^-M1-AQP4-DNA-based CBA, but positive in its M23-DNA-based counterpart [40], were unequivocally positive in both of the two assays evaluated in the present study. Such factors may include differences in secondary antibodies, pre-analytical sample treatment (pre-adsorption with rabbit liver powder as used in reference [40] versus no pre-adsorption in the present study), starting dilutions, use of tagged versus untagged AQP4, differences in expression levels *etc*.

In line with our findings, many assays employing M1-AQP4-DNA in the past have yielded high sensitivity rates and previous, smaller studies have reported equivalence or no difference between M1 and M23 assays [28]. A recent ELISA study reported similar positivity rates between denatured M23-AQP4 and denatured M1-AQP4 though the average OD value was approximately 20% higher with AQP4-M23 [31]. Similarly, no increase in sensitivity rates was found on direct comparison between an M1-AQP4-based and an M1 + M23-AQP4-based fluorescence-based immunoprecipitation assay [32] as well as in a FACS study [42]. Finally, a recent study found no substantial M23 expression in M1-transfected HEK293 cells [43] and two studies suggested binding of AQP4-IgG-positive samples to both M1 and M23 tetramers, as well as to M1, in the absence of high-order arrays [43,44]. Unfortunately, the exact vector sequences used in some of those studies were not reported, which makes it impossible to decide whether LS played a role or not.

However, investigations of the molecular mechanisms underlying the differential performance of the various assays available were not the objective of the present study, which focuses on the practical, that is, clinical, value of the two assays evaluated here.

Although we found only a single sample in the present cohort that was positive for AQP4-Ab only in the M23-DNA-based assay, the generally higher signal intensity observed with that substrate resulted in a lower number of samples classified as very weakly positive. Such samples can well pose a challenge to less specialized assessors in routine laboratories, and higher signal intensity would facilitate diagnosis in those cases. If it would be possible to improve the signal-to-noise ratio of the M23-based assay evaluated here without reducing sensitivity, that assay could be an important step forward.

In the present study, formalin-fixed cells were used. A previous study suggested that use of live cells might further improve AQP4-IgG detection rates [32]. However, the lack of a sufficiently large control cohort in that study makes it difficult to appreciate fully the specificity of the respective live-cell assay and, thus, to decide whether all of the reported additional positives were true positives. Of note, the previous C^-3^-M1-AQP4-DNA-based CBA [40] that had yielded a negative result in two samples clearly positive in the present fixed-cell C^-3^-M1-AQP4-DNA-based CBA (and in two independent M23-DNA-based assays) had been a live-cell assay, indicating that other factors may compensate for the use of fixed cells. Moreover, live-cell assays have a number of potential disadvantages: First, live-cell assays are not exactly standardizable because cells have to be newly transfected before each run and transfection rates may thus vary over time; this potentially prevents their usability for long-term monitoring. Stably transfected cells might be advantageous in this regard; however, expression rates may still drop to some extent and thus need to be monitored over time. Second, live-cell assays require cell culture facilities and highly specialized personal and are thus available only at very few specialized laboratories worldwide. Third, compared to commercially available, ready-to-use fixed cell assays, live-cell assays are usually more time- and labor-consuming and thus less suitable for routine laboratories not focusing on AQP4-IgG testing. While live-cell assays may prove useful in the context of scientific studies, standardized assays that can be made readily available to all laboratories providing routine testing for autoantibodies are important for everyday clinical practice.

In summary, the present study argues against an urgent need to substitute the currently widely used C^-3^-M1-AQP4-DNA-based CBA with LS first described by us in 2010 with an M23-AQP4-DNA-based CBA and, importantly, largely affirms the validity of the numerous studies that used that assay for assessing the frequency of NMO-IgG/AQP4-IgG seropositivity in NMO spectrum disorders (for example, [12,22,26,27,45-50]). While this particular M1-DNA-based assay showed similar positivity rates to an M23-DNA-based assay, it should be underlined that this does not imply general equivalence between M1- and M23-DNA-based assays. LS and other factors specific to this particular assay may have played an important role. This should be considered when it comes to developing future assays for detecting AQP4-IgG based on full-length AQP4-DNA. The higher signal intensity observed in the M23 cells could be especially advantageous when it comes to detecting low AQP4-IgG titers in patients under immunosuppressive treatment and might thus facilitate long-term monitoring of AQP4-IgG titers in such patients. Finally, the worse signal-to-noise ratio observed with some samples in the M23 assay, the cause of which is unknown, might limit the diagnostic value of the M23-based CBA evaluated here and warrants further endeavors to optimize that assay.

## Abbreviations

CBA: cell-based assay; CNS: central nervous system; FI: fluorescence intensity; LS: leaky scanning; NMO: neuromyelitis optica; OAPs: orthogonal arrays of particles; ON: optic neuritis.

## Competing interests

K.F. is an employee of Euroimmun, Luebeck, Germany. The work of S.J. was supported by research grants from the European Committee for Treatment and Research in Multiple Sclerosis (ECTRIMS). Fr.P. and Fl.P. are supported by the German Research Foundation (DFG Exc 257) and the German Ministry of Education and Research (Competence Network Multiple Sclerosis). O.A. is supported by the German Research Foundation (DFG SFB974, GRK1033), German Ministry for Education and Research (EDEN, EU-FP7), Schaufler Foundation, and the Walter- and Ilse-Rose-Foundation. The work of B.W. was supported by a research grant from Merck Serono and from the Dietmar Hopp Stiftung. The other authors report no conflicts of interests.

## Authors' contributions

SJ and BW conceived the study; SJ tested the samples, analyzed the data, and wrote the initial draft; FP, KR, OA, IK, DF, MR, FP, and BW were involved in patient care; KF was involved in preparation of the test substrates; all authors were involved in critically revising the manuscript for important intellectual content. All authors read and approved the final manuscript.

## References

[B1] Jarius S Ruprecht K Wildemann B Kuempfel T Ringelstein M Geis C Kleiter I Kleinschnitz C Berthele A Brettschneider J Hellwig K Hemmer B Linker RA Lauda F Mayer CA Tumani H Melms A Trebst C Stangel M Marziniak M Hoffmann F Schippling S Faiss JH Neuhaus O Ettrich B Zentner C Guthke K Hofstadt-van Oy U Reuss R Pellkofer H Contrasting disease patterns in seropositive and seronegative neuromyelitis optica: A multicentre study of 175 patients J Neuroinflammation 2012 9 14 22260418 10.1186/1742-2094-9-14 PMC3283476

[B2] Trebst C Jarius S Berthele A Paul F Schippling S Wildemann B Borisow N Kleiter I Aktas O Kumpfel T Update on the diagnosis and treatment of neuromyelitis optica: Recommendations of the Neuromyelitis Optica Study Group (NEMOS) J Neurol 2013 261 1 16 24272588 10.1007/s00415-013-7169-7 PMC3895189

[B3] Wildemann B Jarius S Paul F Neuromyelitis optica Nervenarzt 2013 84 436 441 23306312 10.1007/s00115-012-3602-x

[B4] Jarius S Wildemann B The history of neuromyelitis optica J Neuroinflammation 2013 10 8 23320783 10.1186/1742-2094-10-8 PMC3599417

[B5] Lennon VA Wingerchuk DM Kryzer TJ Pittock SJ Lucchinetti CF Fujihara K Nakashima I Weinshenker BG A serum autoantibody marker of neuromyelitis optica: distinction from multiple sclerosis Lancet 2004 364 2106 2112 15589308 10.1016/S0140-6736(04)17551-X

[B6] Jarius S Franciotta D Bergamaschi R Wright H Littleton E Palace J Hohlfeld R Vincent A NMO-IgG in the diagnosis of neuromyelitis optica Neurology 2007 68 1076 1077 17287449 10.1212/01.wnl.0000256822.01222.bd

[B7] Lennon VA Kryzer TJ Pittock SJ Verkman AS Hinson SR IgG marker of optic-spinal multiple sclerosis binds to the aquaporin-4 water channel J Exp Med 2005 202 473 477 16087714 10.1084/jem.20050304 PMC2212860

[B8] Paul F Jarius S Aktas O Bluthner M Bauer O Appelhans H Franciotta D Bergamaschi R Littleton E Palace J Seelig HP Hohlfeld R Vincent A Zipp F Antibody to aquaporin 4 in the diagnosis of neuromyelitis optica PLoS Med 2007 4 e133 17439296 10.1371/journal.pmed.0040133 PMC1852124

[B9] Jarius S Paul F Franciotta D Waters P Zipp F Hohlfeld R Vincent A Wildemann B Mechanisms of disease: aquaporin-4 antibodies in neuromyelitis optica Nat Clin Pract Neurol 2008 4 202 214 18334978 10.1038/ncpneuro0764

[B10] Jarius S Wildemann B AQP4 antibodies in neuromyelitis optica: diagnostic and pathogenetic relevance Nat Rev Neurol 2010 6 383 392 20639914 10.1038/nrneurol.2010.72

[B11] Jarius S Aboul-Enein F Waters P Kuenz B Hauser A Berger T Lang W Reindl M Vincent A Kristoferitsch W Antibody to aquaporin-4 in the long-term course of neuromyelitis optica Brain 2008 131 3072 3080 18945724 10.1093/brain/awn240 PMC2577801

[B12] Levy M Wildemann B Jarius S Orellano B Sasidharan S Weber MS Stuve O Immunopathogenesis of neuromyelitis optica Adv Immunol 2014 121 213 242 24388217 10.1016/B978-0-12-800100-4.00006-4

[B13] Jarius S Wildemann B Paul F Neuromyelitis optica: clinical features, immunopathogenesis and treatment Clin Exp Immunol 2014 176 149 164 24666204 10.1111/cei.12271 PMC3992027

[B14] Wildemann B Jarius S The expanding range of autoimmune disorders of the nervous system Lancet Neurol 2013 12 22 24 23237898 10.1016/S1474-4422(12)70301-0

[B15] Wildemann B Bien CG Immune-mediated encephalomyelitis Nervenarzt 2013 84 435 23525588 10.1007/s00115-012-3601-y

[B16] Lai M Huijbers MG Lancaster E Graus F Bataller L Balice-Gordon R Cowell JK Dalmau J Investigation of LGI1 as the antigen in limbic encephalitis previously attributed to potassium channels: a case series Lancet Neurol 2010 9 776 785 20580615 10.1016/S1474-4422(10)70137-X PMC3086669

[B17] Reindl M Di Pauli F Rostasy K Berger T The spectrum of MOG autoantibody-associated demyelinating diseases Nat Rev Neurol 2013 9 455 461 23797245 10.1038/nrneurol.2013.118

[B18] Lancaster E Lai M Peng X Hughes E Constantinescu R Raizer J Friedman D Skeen MB Grisold W Kimura A Ohta K Iizuka T Guzman M Graus F Moss SJ Balice-Gordon R Dalmau J Antibodies to the GABA(B) receptor in limbic encephalitis with seizures: case series and characterisation of the antigen Lancet Neurol 2010 9 67 76 19962348 10.1016/S1474-4422(09)70324-2 PMC2824142

[B19] Jarius S Wandinger KP Horn S Heuer H Wildemann B A new Purkinje cell antibody (anti-Ca) associated with subacute cerebellar ataxia: immunological characterization J Neuroinflammation 2010 7 21 20226058 10.1186/1742-2094-7-21 PMC2848133

[B20] Irani SR Alexander S Waters P Kleopa KA Pettingill P Zuliani L Peles E Buckley C Lang B Vincent A Antibodies to Kv1 potassium channel-complex proteins leucine-rich, glioma inactivated 1 protein and contactin-associated protein-2 in limbic encephalitis, Morvan’s syndrome and acquired neuromyotonia Brain 2010 133 2734 2748 20663977 10.1093/brain/awq213 PMC2929337

[B21] Lai M Hughes EG Peng X Zhou L Gleichman AJ Shu H Mata S Kremens D Vitaliani R Geschwind MD Bataller L Kalb RG Davis R Graus F Lynch DR Balice-Gordon R Dalmau J AMPA receptor antibodies in limbic encephalitis alter synaptic receptor location Ann Neurol 2009 65 424 434 19338055 10.1002/ana.21589 PMC2677127

[B22] Jarius S Frederikson J Waters P Paul F Akman-Demir G Marignier R Franciotta D Ruprecht K Kuenz B Rommer P Kristoferitsch W Wildemann B Vincent A Frequency and prognostic impact of antibodies to aquaporin-4 in patients with optic neuritis J Neurol Sci 2010 298 158 162 20850793 10.1016/j.jns.2010.07.011

[B23] Matiello M Lennon VA Jacob A Pittock SJ Lucchinetti CF Wingerchuk DM Weinshenker BG NMO-IgG predicts the outcome of recurrent optic neuritis Neurology 2008 70 2197 2200 18434643 10.1212/01.wnl.0000303817.82134.da

[B24] Petzold A Pittock S Lennon V Maggiore C Weinshenker BG Plant GT Neuromyelitis optica-IgG (aquaporin-4) autoantibodies in immune mediated optic neuritis J Neurol Neurosurg Psychiatry 2010 81 109 111 20019228 10.1136/jnnp.2008.146894

[B25] Akman-Demir G Tuzun E Waters P Icoz S Kurtuncu M Jarius S Yapici Z Mutlu M Yesilot N Vincent A Eraksoy M Prognostic implications of aquaporin-4 antibody status in neuromyelitis optica patients J Neurol 2011 258 464 470 20953960 10.1007/s00415-010-5780-4

[B26] Jarius S Probst C Borowski K Franciotta D Wildemann B Stoecker W Wandinger KP Standardized method for the detection of antibodies to aquaporin-4 based on a highly sensitive immunofluorescence assay employing recombinant target antigen J Neurol Sci 2010 291 52 56 20117794 10.1016/j.jns.2010.01.002

[B27] Jarius S Franciotta D Paul F Bergamaschi R Rommer PS Ruprecht K Ringelstein M Aktas O Kristoferitsch W Wildemann B Testing for antibodies to human aquaporin-4 by ELISA: Sensitivity, specificity, and direct comparison with immunohistochemistry J Neurol Sci 2012 320 32 37 22705047 10.1016/j.jns.2012.06.002

[B28] Jarius S Wildemann B Aquaporin-4 antibodies (NMO-IgG) as a serological marker of neuromyelitis optica: a critical review of the literature Brain Pathol 2013 23 661 683 24118483 10.1111/bpa.12084 PMC8028894

[B29] Waters P Jarius S Littleton E Leite MI Jacob S Gray B Geraldes R Vale T Jacob A Palace J Maxwell S Beeson D Vincent A Aquaporin-4 antibodies in neuromyelitis optica and longitudinally extensive transverse myelitis Arch Neurol 2008 65 913 919 18625857 10.1001/archneur.65.7.913

[B30] Waters P Pittock SJ Bennett JL Jarius S Weinshenker BG Wingerchuk DM Evaluation of aquaporin-4 antibody assays Clin Exp Neuroimmunol 2014 doi:10.1111/cen3.12107 10.1111/cen3.12107 PMC5102503 27840658

[B31] Kim W Lee JE Li XF Kim SH Han BG Lee BI Kim JK Choi K Kim HJ Quantitative measurement of anti-aquaporin-4 antibodies by enzyme-linked immunosorbent assay using purified recombinant human aquaporin-4 Mult Scler 2012 18 578 586 21965418 10.1177/1352458511424590

[B32] Waters PJ McKeon A Leite MI Rajasekharan S Lennon VA Villalobos A Palace J Mandrekar JN Vincent A Bar-Or A Pittock SJ Serologic diagnosis of NMO: a multicenter comparison of aquaporin-4-IgG assays Neurology 2012 78 665 671 discussion 669 22302543 10.1212/WNL.0b013e318248dec1 PMC3286228

[B33] Takahashi T Fujihara K Nakashima I Misu T Miyazawa I Nakamura M Watanabe S Shiga Y Kanaoka C Fujimori J Sato S Itoyama Y Anti-aquaporin-4 antibody is involved in the pathogenesis of NMO: a study on antibody titre Brain 2007 130 1235 1243 17449477 10.1093/brain/awm062

[B34] Nicchia GP Mastrototaro M Rossi A Pisani F Tortorella C Ruggieri M Lia A Trojano M Frigeri A Svelto M Aquaporin-4 orthogonal arrays of particles are the target for neuromyelitis optica autoantibodies Glia 2009 57 1363 1373 19229993 10.1002/glia.20855

[B35] Crane JM Lam C Rossi A Gupta T Bennett JL Verkman AS Binding affinity and specificity of neuromyelitis optica autoantibodies to aquaporin-4 M1/M23 isoforms and orthogonal arrays J Biol Chem 2011 286 16516 16524 21454592 10.1074/jbc.M111.227298 PMC3091256

[B36] Marnetto F Granieri L Sala A Frau J Patanella AK Gilli F Capobianco M Wandinger KP Bertolotto A Validation of a multi-parametric immunofluorescence assay for the detection of anti-AQP4 antibodies in the diagnosis of neuromyelitis optica Mult Scler 2009 15 P154

[B37] Granieri L Marnetto F Valentino P Frau J Patanella AK Nytrova P Sola P Capobianco M Jarius S Bertolotto A Evaluation of a multiparametric immunofluorescence assay for standardization of neuromyelitis optica serology PLoS ONE 2012 7 e38896 22719979 10.1371/journal.pone.0038896 PMC3373605

[B38] Pisani F Sparaneo A Tortorella C Ruggieri M Trojano M Mola MG Nicchia GP Frigeri A Svelto M Aquaporin-4 autoantibodies in Neuromyelitis Optica: AQP4 isoform-dependent sensitivity and specificity PLoS ONE 2013 8 e79185 24260168 10.1371/journal.pone.0079185 PMC3829826

[B39] Rossi A Pisani F Nicchia GP Svelto M Frigeri A Evidences for a leaky scanning mechanism for the synthesis of the shorter M23 protein isoform of aquaporin-4: implication in orthogonal array formation and neuromyelitis optica antibody interaction J Biol Chem 2010 285 4562 4569 20007705 10.1074/jbc.M109.069245 PMC2836061

[B40] Mader S Lutterotti A Di Pauli F Kuenz B Schanda K Aboul-Enein F Khalil M Storch MK Jarius S Kristoferitsch W Berger T Reindl M Patterns of antibody binding to aquaporin-4 isoforms in neuromyelitis optica PLoS ONE 2010 5 e10455 20463974 10.1371/journal.pone.0010455 PMC2864757

[B41] Jiao Y Fryer JP Lennon VA Jenkins SM Quek AM Smith CY McKeon A Costanzi C Iorio R Weinshenker BG Wingerchuk DM Shuster EA Lucchinetti CF Pittock SJ Updated estimate of AQP4-IgG serostatus and disability outcome in neuromyelitis optica Neurology 2013 81 1197 1204 23997151 10.1212/WNL.0b013e3182a6cb5c PMC3795610

[B42] Kalluri SR Illes Z Srivastava R Cree B Menge T Bennett JL Berthele A Hemmer B Quantification and functional characterization of antibodies to native aquaporin 4 in neuromyelitis optica Arch Neurol 2010 67 1201 1208 20937947 10.1001/archneurol.2010.269

[B43] Iorio R Fryer JP Hinson SR Fallier-Becker P Wolburg H Pittock SJ Lennon VA Astrocytic autoantibody of neuromyelitis optica (NMO-IgG) binds to aquaporin-4 extracellular loops, monomers, tetramers and high order arrays J Autoimmun 2013 40 21 27 22906356 10.1016/j.jaut.2012.07.008 PMC3509259

[B44] Crane JM Bennett JL Verkman AS Live cell analysis of aquaporin-4 M1/M23 interactions and regulated orthogonal array assembly in glial cells J Biol Chem 2009 284 35850 35860 19843522 10.1074/jbc.M109.071670 PMC2791014

[B45] Jarius S Jacobi C De Seze J Zephir H Paul F Franciotta D Rommer P Mader S Kleiter I Reindl M Akman-Demir G Seifert-Held T Kristoferitsch W Melms A Wandinger KP Wildemann B Frequency and syndrome specificity of antibodies to aquaporin-4 in neurological patients with rheumatic disorders Mult Scler 2011 17 1067 1073 21543553 10.1177/1352458511403958

[B46] Zavada J Nytrova P Wandinger KP Jarius S Svobodova R Probst C Peterova V Tegzova D Pavelka K Vencovsky J Seroprevalence and specificity of NMO-IgG (anti-aquaporin 4 antibodies) in patients with neuropsychiatric systemic lupus erythematosus Rheumatol Int 2013 33 259 263 22038193 10.1007/s00296-011-2176-4

[B47] Von Glehn F Jarius S Penalva De Oliveira AC Brandao CO Farias AS Damasceno A Casseb J Moraes AS Longhini AL Wandinger KP Damasceno BP Wildemann B Santos LM Aquaporin-4 antibodies are not related to HTLV-1 associated myelopathy PLoS ONE 2012 7 e39372 22808032 10.1371/journal.pone.0039372 PMC3393709

[B48] Jarius S Paul F Franciotta D De Seze J Munch C Salvetti M Ruprecht K Liebetrau M Wandinger KP Akman-Demir G Melms A Kristoferitsch W Wildemann B Neuromyelitis optica spectrum disorders in patients with myasthenia gravis: ten new aquaporin-4 antibody positive cases and a review of the literature Mult Scler 2012 18 1135 1143 22183934 10.1177/1352458511431728

[B49] Wandinger KP Stangel M Witte T Venables P Charles P Jarius S Wildemann B Probst C Iking-Konert C Schneider M Autoantibodies against aquaporin-4 in patients with neuropsychiatric systemic lupus erythematosus and primary Sjogren’s syndrome Arthritis Rheum 2010 62 1198 1200 20131265 10.1002/art.27337

[B50] Jarius S Franciotta D Paul F Ruprecht K Bergamaschi R Rommer PS Reuss R Probst C Kristoferitsch W Wandinger KP Wildemann B Cerebrospinal fluid antibodies to aquaporin-4 in neuromyelitis optica and related disorders: frequency, origin, and diagnostic relevance J Neuroinflammation 2010 7 52 20825655 10.1186/1742-2094-7-52 PMC2945323

